# Prevalence of mental health effects among healthcare professionals during the COVID-19 pandemic

**DOI:** 10.1192/j.eurpsy.2023.1148

**Published:** 2023-07-19

**Authors:** M. Bakola, K. S. Kitsou, C. Kalogirou, N. Vaitsis, S. Aggelakou-Vaitsi, K. Argyropoulos, M. Kampouraki, E. Gkatsi, K. Tsolaki, M. Vakas, A. Theochari, K. Mavridou, S. Karatzeni, M. Siali, X. Bazoukis, M. Chalkidou, V. Karagianni, P. Gourzis, E. Jelastopulu

**Affiliations:** 1 Department of Public Health; 2 Department of Psychiatry, University of Patras, Medical School, Patras, Greece

## Abstract

**Introduction:**

The COVID-19 pandemic has placed extraordinary mental health burdens on healthcare professionals. For women, it is a major challenge to reconcile the diverse roles of a professional, mother, and wife. The COVID-19 pandemic has exacerbated this, increasing their vulnerability to mental health issues.

**Objectives:**

The aim of the study was to assess COVID-19-related mental health of healthcare professionals and to investigate whether possible gender differences as well as other parameters are associated with mental health disturbances.

**Methods:**

We conducted a nationwide cross-sectional study of healthcare professionals working in hospitals or primary care settings in Greece from April to June 2022. Participants answered a questionnaire that included socio-demographic and other parameters, the Coronavirus Anxiety Scale (CAS), the *Coronavirus* Reassurance-Seeking Behaviors Scale (CRBS), and the Obsession with COVID-19 scale (OCS).

**Results:**

A total of 464 healthcare professionals participated in the study, 71.2% were females and two-thirds were 31-50 years old. Elevated levels of anxiety, frequent reassurance seeking activities and persistent troubling thoughts related to COVID-19 were found in 5.8%, 3.2% and 6.1%, respectively. However, females reported significant higher mean levels on CAS and CRBS compared to males (2.41 vs 1.60, p=0.015, and 3.36 vs 2.64, p=0.041, respectively). Participants living in smaller areas had increased levels on all three scales (CAS, p < 0.001; CRBS, p = 0.007; OCS, p < 0.001), indicating thus higher coronaphobia, more frequent reassurance-seeking behaviors and disturbed thinking about COVID-19, compared to healthcare workers living in urban regions. Furthermore, lower educational level is also associated with higher values on CAS, CRBS and OCS (p < 0.003; p = 0.017; p < 0.023, respectively). Nurses experience higher anxiety scores (2.96) than physicians (1.92, p=0.013) or other healthcare workers (1.87, p=0.016). No dysfunctional thinking about COVID-19 is observed in medical doctors, whereas nurses and other healthcare workers experience higher levels on OCS.

**Conclusions:**

Our study does not show any worrying increased psychological dysfunction related to COVID-19 pandemic among healthcare workers in general. However, females have increased levels than males. Thus, support and mental health protecting strategies should be applied primarily to female healthcare professionals when necessary.

**Disclosure of Interest:**

None Declared

